# Drug Shortage: Causes, Impact, and Mitigation Strategies

**DOI:** 10.3389/fphar.2021.693426

**Published:** 2021-07-09

**Authors:** Sundus Shukar, Fatima Zahoor, Khezar Hayat, Amna Saeed, Ali Hassan Gillani, Sumaira Omer, Shuchen Hu, Zaheer-Ud-Din Babar, Yu Fang, Caijun Yang

**Affiliations:** ^1^ Department of Pharmacy Administration and Clinical Pharmacy, School of Pharmacy, Xi’an Jiaotong University, Xi’an, China; ^2^ Center for Drug Safety and Policy Research, Xian Jiaotong University, Xi’an, China; ^3^ Shaanxi Centre for Health Reform and Development Research, Xi’an, China; ^4^ Research Institute for Drug Safety and Monitoring, Institute of Pharmaceutical Science and Technology, China’s Western Technological Innovation Harbor, Xi’an, China; ^5^ Department of Pharmacy, Quaid-i-Azam University, Islamabad, Pakistan; ^6^ Yusra Institute of Pharmaceutical Sciences, Islamabad, Pakistan; ^7^ Institute of Pharmaceutical Sciences, University of Veterinary and Animal Sciences, Lahore, Pakistan; ^8^ Department of Pharmacy, School of Applied Sciences, University of Huddersfield, Huddersfield, United Kingdom

**Keywords:** medicine shortage, drug shortage, definitions, causes, impacts, mitigation strategies

## Abstract

Drug shortage is a global issue affecting low, middle, and high-income countries. Many countries have developed various strategies to overcome the problem, while the problem is accelerating, affecting the whole world. All types of drugs, such as essential life-saving drugs, oncology medicines, antimicrobial drugs, analgesics, opioids, cardiovascular drugs, radiopharmaceutical, and parenteral products, are liable to the shortage. Among all pharmaceutical dosage forms, sterile injectable products have a higher risk of shortage than other forms. The causes of shortage are multifactorial, including supply issues, demand issues, and regulatory issues. Supply issues consist of manufacturing problems, unavailability of raw materials, logistic problems, and business problems. In contrast, demand issues include just-in-time inventory, higher demand for a product, seasonal demand, and unpredictable demand. For regulatory issues, one important factor is the lack of a unified definition of drug shortage. Drug shortage affects all stakeholders from economic, clinical, and humanistic aspects. WHO established global mitigation strategies from four levels to overcome drug shortages globally. It includes a workaround to tackle the current shortage, operational improvements to reduce the shortage risk and achieve early warning, changes in governmental policies, and education and training of all health professionals about managing shortages.

## Introduction

Medicines are vital elements to health-care, and access to medicines is a fundamental human right (Hogerzeil, 2006). The World Health Organization (WHO) defines essential medicines that “satisfy the population’s priority health care needs” (De Weerdt et al., 2015). However, the prevailing drug shortage problems bring significant challenges to the health care system.

**FIGURE 1:**
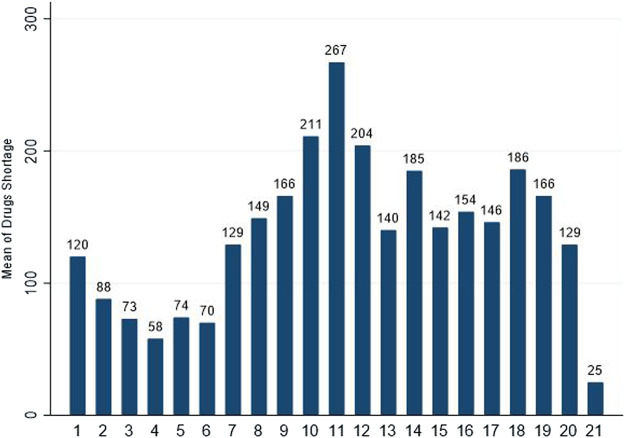
Number of drugs in shortage per year reported by University of Utah Drug Information Services (UUDIS) (American Society of Health System Pharmacists, 2020, June 30).

The shortage of drugs remained a problem in history up to the present date. The first time of drug scarcity in the record could be traced back to the insulin shortage in the early 1920s. Since then, the drug shortage is more common worldwide (Yang et al., 2016; Walker et al., 2017; Unguru et al., 2019; Zwaida et al., 2019). In 2012, Gray and Manasse found that 21 countries were affected by drug shortages (Russell et al., 2017), and the recent data of the University of Utah Drug Information Services reported 129 medicine shortages in the United States in 2020, as shown in Figure 1 (American Society of Health System Pharmacists, 2020, june 30).

Drug shortage has prevailed throughout the globe affecting high, middle, and low-income countries. In high-income countries, the drug shortage has been in excessive focus compared to other regions. The causes include manufacturing problems, business decisions, raw material unavailability, and regulatory issues (Ventola, 2011). Many agencies, associations, and governments have developed different policies, programs, research studies, and guidelines to address this event (Ferrario et al., 2017; Bochenek et al., 2018). However, drug shortage still causes severe health and economic crises (Dill and Ahn, 2014; Dave et al., 2018). Unlike high-income countries, low-middle income countries (LMICs) have several new causes for drug shortage, including licensing of manufacturers/products, shortage of raw material for a local manufacturer, drug smuggling, and lodging tax government policies (Khan, 2019). Research studies in these countries found a need for government strategies to build research information platforms (Yang et al., 2016) to mitigate this issue (Walker et al., 2017; Atif et al., 2019). Low-income countries have few research studies and lack policies to deal with this event. A few studies reported a stock-out of essential medicines in Malawi, Egypt, and Uganda (Alsirafy and Farag, 2016; Khuluza and Heide, 2017). Literature also reported shortages of TB drugs, Ketamine, and drugs against HIV in African countries (Seunanden and Day, 2013; Gray, 2014; Addo et al., 2018; Wall and Bangalee, 2019).

Drug shortages have diverse impacts on different stakeholders, especially patients. Patients are facing issues such as increased patient monitoring, suboptimal treatment through the use of alternative drugs, delayed care, being transferred to other institutions, increased length of hospitalization, readmission due to adverse events/treatment failure/relapse, associated care cancellations (surgery: bone marrow transplantation), or even death (Mclaughlin et al., 2013; Rider et al., 2013; Souliotis et al., 2014; Blaine et al., 2016; Yang et al., 2016; Alruthia et al., 2017). Irrespective of the nature of the cause, the effects of the drug shortage are so endless that most of the professionals in health institutions are affected and well aware of its long-lasting drastic impact, reported by the survey performed by the European Association of Hospital Pharmacists (EAHP) among 27 countries (Dill and Ahn, 2014; Tan et al., 2016; Haider et al., 2019; Phuong et al., 2019).

Different countries have developed various strategies to address the drug shortage. These strategies vary depending upon the financial condition, the health system strength, and research studies, such as increased reporting systems, changes in policies, drug shortage platforms, and accelerated drug approval (Thoma et al., 2014; Yang et al., 2016). Some authorities evaluate drug shortages and deliver guidelines directly to health stakeholders (Ferrario et al., 2017; Bochenek et al., 2018), while some hospitals purchase extra inventory as a buffer stock to prevent shortages (Fox et al., 2014). Some regulatory authorities establish platforms for tracking and reporting shortages (Quilty, 2014), and some countries are trying to strengthen their food and drug institutions (Alruthia et al., 2018; ASHP and Healthcare, September 30, 2020). As reported by the FDA from 117 (2012) to 23 (2016), the decrease in drug shortage showed that the US’s approaches have worked to some degree. However, the issue is still playing with the healthcare system and patients, as seen with 98 (2020) drug shortages (ASHP and Healthcare, 30/09/2020; Food, 2018; Fox and McLaughlin, 2018; Phuong et al., 2019). Data from low and middle-income countries is not enough to compare them with high-income countries. Similarly, the US has more data in comparison (Schwartzberg et al., 2017).

The drug shortage problem has severe aggressive impacts on the health care system and public health. This review aims to evaluate the current drug shortage situation in high, middle, and low-income countries and summarize its causes, impacts, and mitigation strategies that the world can implement to overcome.

## Definition of Drug Shortage

### Lack of Standardized Definition

There is a lack of a standardized definition of drug shortage globally. The definition varies from one regulatory authority to another. Some authorities define the drug shortage from the supply side, and some define it from the demand side. Some authorities define it according to their level in the drug supply chain (whether it’s low supply or increased demand), and some define it concerning the timeframe or duration, for example, inability to dispense to a patient in a specific time (Videau et al., 2019a). A study in the EU reported 26 unique definitions 2), and WHO found 56 definitions worldwide. Three possible reasons could contribute to the above event. First, different authorities representing various stakeholders define drug shortages according to different criteria. Second, there is a lack of high-quality scientific research on drug shortage to provide a widely accepted definition (Kaakeh et al., 2011; Klobuchar, 2011; Bochenek et al., 2018). Third, a lack of transparent quantitative data also impedes a global definition of shortage (Bogaert et al., 2015; Meloni et al., 2017). The standard global definition is needed because different definitions used by different countries, which define medicine shortages at different scales, make it impossible to estimate and analyze medicine shortages at the international level (Bochenek et al., 2018).

Fortunately, the WHO and the EU are working on this problem. In 2016, WHO convened an informal consultation of experts to develop technical definitions of shortages and stock-outs of medicines and vaccines. In the meeting, they analyzed those 56 definitions used for drug shortages. Terms were mapped to different supply chain areas, from manufacturing to dispensing to patients, and then the mapped terms were grouped according to whether they were related to supply or demand. Finally, two draft definitions, one from the supply-side and the other from the demand-side, were developed by consensus and some additional notes for future refinement of the drugs shortage definition (Organization, 2017). On the supply-side, a shortage occurs “when the supply of medicines, health products, and vaccines identified as essential by the health system is considered to be insufficient to meet public health and patient needs.” While on the demand-side, a shortage occurs “when demand exceeds supply at any point in the supply chain and may ultimately create a stock-out at the point of appropriate service delivery to the patient if the cause of the shortage cannot be resolved promptly relative to the clinical needs of the patient.” Usually, shortages on the demand-side may not be found shortages on the supply-side, while the supply-side shortage would ultimately cause a shortage on the demand-side. And in 2019, the EMA and HMA joint task force released the first harmonized “shortage” definition for all the EU countries, stated as “a shortage of a medicinal product for human and veterinary use occurs when supply does not meet demand at a national level” (Musazzi et al., 2020). Comparatively, the EU’s definition of shortage makes the international comparison feasible. While the two definitions proposed by WHO gives the stakeholders a standard from the demand or supply-side to define and manage their shortages.

### Widely Used Definitions

There are several definitions widely used by researchers, including definitions given by the University of Utah Drug Information Service (UUDIS), the American Society of Health-System Pharmacists (ASHP) (Fox et al., 2014; Phuong et al., 2019), the United States Food and Drug (Administration US FDA, 2020), the Health Canada (Dill and Ahn, 2014), the International Society of Pharmaceutical Engineering (ISPE), the European Federation of Pharmaceutical Industries and Associations (EFPIA) (Fox and Tyler, 2013) and the International Pharmaceutical Federation (FIP).

University of Utah Drug Information Services (UUDIS) and American Society of Hospital Pharmacists (ASHP) define drug shortage as “a supply issue that affects how the pharmacy prepares or dispenses a drug product or influences patient care when prescribers must use an alternate agent” (Fox et al., 2014; Phuong et al., 2019). The United States FDA has given three definitions. The first one defines the medicine shortage from the supply side as “a period of time when the demand or projected demand for drug exceeds the supply of drug” (Phuong et al., 2019), and the second one defines it from the demand side as “a shortage will occur when demands exceeds supply at any point in the supply chain may ultimately create a “stock-out” at the point of appropriate service delivery to the patient if the cause of shortage cannot be resolved in a timely manner relative to the clinical needs of the patients” (Acosta et al., 2019). The third one defines drug shortage as “a situation in which the total supply of all clinically interchangeable versions of an FDA regulated drug product is inadequate to meet the projected demand at the user level” (Schwartzberg et al., 2017). Health Canada describes a drug shortage as “when a manufacturer/importer anticipates that they cannot supply a drug to meet projected demand” (Dill and Ahn, 2014). ISPE defines drug shortage as “a situation in which total supply of an approved medicine is inadequate to meet the current projected demand at the user level.” EFPIA defines medication shortage as “a crisis situation caused by any ability of any Market Authorization Holder (MAH) to supply a medicine with a specific API to market over an extended period of time resulting in the unavailability of this medication for patients” (Fox et al., 2014). The International Pharmaceutical Federation (FIP) has defined medicines shortages as “A drug supply issue requiring a change. It impacts patient care and requires the use of alternative agents” (FIP), September 14, 2020).

Moreover, the low and middle-income countries have the absence of an official definition. Middle-income countries like Iran (Setayesh and Mackey, 2016), Iraq (Acosta et al., 2019), Jardon (Zu’bi and Abdallah, 2016), Egypt (Abdelrahman et al., 2016), China (Acosta et al., 2019) used definitions of United States FDA, ASHP, and FIP in their research studies. Similarly, low-income countries like Pakistan and Kenya do not have their official definition (Fatima and Khaliq, 2017; Atif et al., 2019). These definitions are widely used but vary from each other based on some parameters, give different reporting criteria, and do not provide the exact estimation of drug shortage (Bochenek et al., 2018). It is evident from the different number of products reported as shortages by the FDA and ASHP simultaneously, due to different reporting systems’ characteristics and different definitions (Alsheikh et al., 2016).

## Drugs Reported in Shortage

Nearly all types of drugs have been reported in shortage, including antibiotics (A07AA/D01AA/G01AA/J02AA/S01AA), antiretroviral drugs (J05AR), anti-protozoal (P01), antineoplastic agents (L01), cardiovascular medicines (C), analgesics (N02), etc. Different countries or areas encounter different drugs in shortage depending upon health conditions (Mazer-Amirshahi et al., 2014; Rinaldi et al., 2017). However, essential medicines (Hedman, 2016) and emergency medicines (Dill and Ahn, 2014; Mazer-Amirshahi et al., 2014; Alsirafy and Farag, 2016; Fox and McLaughlin, 2018; Benge and Burka, 2019) are more liable to shortage than other medicines. In high economic countries, research studies found different classes of drugs in short supply. Still, too few studies in low-middle income and low-income countries are found to depict the complete picture; only some research studies focus on the affordability/availability and shortage of some essential medicines (Phuong et al., 2019). Almost all classes of medicines were in short supply in high-income countries. Antimicrobial agents are the most affected class by drug shortage (Mazer-Amirshahi et al., 2017), along with oncology drugs (both chemotherapeutic (D06B/D06C/D06BX) and non-chemotherapeutic drugs) (Woodcock and Wosinska, 2013; Costelloe et al., 2015). Benzathine Penicillin G (J01CE08) shortage occurred in the United States (2014), and the reason reported by ASHP was a delay in manufacturing (Organization, 2016). A study in Iran found 73 cancer drugs in shortage, covering most areas (Setayesh and Mackey, 2016). Many oncology drugs were reported in shortage in the US, for example, Mechlorethamine, Leucovorin (L01BA), Daunorubicin (L01DB02), PEGylated liposomal Doxorubicin (L01DB01), etc. (Phuong et al., 2019). In Canada, the Sandoz crisis (2011) started with slow production, ultimately to the plant’s shut to meet United States FDA standards. It led to the shortage of morphine (N02AA01) used for patients at the end stage of their lives, and Ondansetron is used to relieve nausea during chemotherapy (Videau et al., 2019a). Cardiovascular drugs like Labetalol (C07A G01) and Metoprolol (C07AB02), Methyldopa (C02AB), and pindolol (C07AA03) are found in the United States FDA drug shortage list (Administration UF, 2020). Adrenaline has repeatedly been reported in short supply by Therapeutic Goods Administration Australia (2014, 2020) caused by commercial changes and unexpectedly increased demand-effected critical patients (Organization, 2016). Shortages of analgesics were also reported, such as Salsalate 500 mg (N02BA06) drug in the United States (Dave et al., 2018). Currently, the United States FDA shortage drug list still includes Ketoprefen (M01AE03) (Administration USF.A.D, 2020). A study done in Canada in 2017 on the impacts of Clobazam (N05BA09) (benzodiazepine) shortages on patients with epilepsy found that patients complained about out-of-pocket cost (Phuong et al., 2019). The most affected drugs in the United States in 2016 were antibiotics, followed by electrolytes, chemotherapy medicines, cardiovascular drugs, and CNS agents. But in 2020, analgesics, sedatives, paralytics were short due to their increased demand in COVID-19. Moreover, cardiovascular and CNS agents in injectables were also short (Pharmacists, 2020). On the other hand, a study of EU in 2014 found that CNS drugs were most short, followed by anti-infective drugs, cardiovascular drugs, antineoplastic/immunomodulatory agents, and GIT drugs (Pauwels et al., 2014), but the EAHP survey in 2019 found that antimicrobial agents were on the top with oncology medicines second, followed by anaesthetic agents The shortage of oncology medicines increased in comparison to the data from 2018 EAHP survey (Miljković et al., 2020a).

In middle-income and low-income countries, literature found shortages of essential medicines. Stock-out of antiretroviral drugs (ART) occurred many times in African countries (Meloni et al., 2017). Besides, Deslanoside (C01AA07), Digoxin (C01AA05), Enalapril (C09AA02), Adrenaline, Noradrenaline, Isosorbide dinitrate (C01DA08), and Nifedipine (C08CA05) were reported in shortage in China (Yang et al., 2016). The shortage of antimalarial drugs was seen in many low and middle-income countries. Acute shortage of antimalarial drugs (Artemether (P01BE02)/Lumefantrine (P01BF01) was found in Kenya, Sub-Saharan Africa, and Uganda due to the delayed procurement process which led to an increased mortality rate (Malik et al., 2013). Shortage of Chloroquine and Sulphadoxine/Pyrimethamine (Fansidar) was also found in the public and private sector of Pakistan, creating a gap in effective malaria control (Malik et al., 2013). Shortage of Chloroquine (P01BA01) and Hydroxychloroquine (P01BA02) was found during the COVID-19 pandemic in many countries due to increased demand (Mazer-Amirshahi et al., 2020). In the surge of the COVID-19 pandemic, a shortage of Sertraline (N06AB06) and Midazolam (N05CD08) occurred due to the increased demand for antidepressant drugs (N06A/N06CA) to manage stress (Administration USF.A.D, 2020). In low-income countries, literature cited medicines used for TB, Malaria, and HIV due to the increased incidence of these diseases and the unavailability of essential drugs rather than drug shortages or stock-out. Table 1 shows the medicines affected by shortages in different countries (Elbagir et al., 1995; Kangwana et al., 2009; Tren et al., 2009; Ballinger, 2010; Ventola, 2011; Griffith et al., 2012; Liang and Mackey, 2012; Malik et al., 2013; Quilty, 2014; Ricci et al., 2016; Donohue and Angus, 2017; Gross et al., 2017; Hsueh et al., 2017; Meloni et al., 2017; Tan et al., 2017; Vail et al., 2017; Banerjee et al., 2018; Dave et al., 2018; Gundlapalli et al., 2018; Holcombe et al., 2018; Said et al., 2018; Benge and Burka, 2019; Visage et al., 2019; Yellapu et al., 2019; Benhabib et al., 2020; Choo and Rajkumar, 2020; Clark et al., 2020; Honda et al., 2020; Siow et al., 2020; Sun et al., 2020; Mclaughlin et al., 2017; Dave et al., 2018).

**TABLE 1:** Medicines involved in shortage.

Therapeutic class	Agents involved	ATC code	Countries affected	Reasons for shortage	Impacts
Antibiotics	Ticarcillin/Clauvulanate Quilty (2014)	J01CA13	Australia	Unknown	Use of alternative drugs with greater risk
	Piperacillin-tazobactam Gross et al. (2017), Hsueh et al. (2017)	J01CA12-J01CG02	United States	Manufacturing issues	First-line agent for many nosocomial infections, alternative drugs with greater risk
	Meropenem, imipenem Hsueh et al. (2017), Gundlapalli et al. (2018)	J01DH02 J01DH56	United States	Procurement failure	Compromised treatment due to the use of alternative drugs
	Penicillin G Yellapu et al. (2019)	J01CA	Globally	Single manufacturer, quality issues	First-line agent for neurosyphilis: Use of other antibiotics cause rheumatic heart disease
	Cefotaxime Banerjee et al. (2018)	J01DD01	United States	Price increasing of raw material	Use of alternative drugs
	Sulfamethoxazole trimethoprim Griffith et al. (2012)	J01EE01	United States	Production interruption	First-line treatment for pneumocystis jiroveci pneumonia and *Stenotrophomonas maltophilia* infection
	Cefazolin Honda et al. (2020)	J01DB04	Japan	Contaminated API from Italy	First-line for common infection, shortage led to the use of other antibiotics with related adverse effects and resistance
	Erythromycin ophthalmic ointment	D10AF02	United States	Production interruption, increased demand	Ophthalmic neonatorum prophylaxis, alternative drugs with adverse effects
Anti-retroviral drugs	Acyclovir Mclaughlin et al. (2017)		United States	Multiple reasons	Managed through oral Val-acyclovir with extra efforts of staff for dose calculation
	Nevirapine (NVP), Lamivudine (3TC) Meloni et al.( 2017)	J05AG01 J05AF05	Nigeria	Economic issues	Mutation resulting in drug resistance
Anti-protozoal	Chloroquine, Sulphadoxine/Pyrimethamine Malik et al. (2013)	P01BA01 P01BD01	Pakistan	Multiple reasons	Appropriate alternative drug use
	Pyrimethamine Dave et al. (2018)	P01BD51	United States	Multiple reasons	Prices increased
	Artemether/Lumefantrine Kangwana et al. (2009), Tren et al. (2009), Malik et al. (2013)	P01BF01	Kenya, Uganda, Saharan Africa	Delayed tendering/procurement process, economic issues	Delayed treatment and associated loss
Antineoplastic agents	Melphalan Said et al. (2018)		Germany	Unknown	Alternate drug
	Cytarabine Ventola (2011)	L01BC01	United States	Raw material shortage + quality defects	Compromised and delayed treatment
Cardiovascular medicines	Metoprolol (beta-blocker) Said et al. (2018)	C07AB02	Germany	Unknown	Compromised treatment
	Digoxin, pyrimethamine Dave et al. (2018)	C01AA05 P01BD51	United States	Unknown	Prices increase
	Abciximab Clark et al. (2020)	B01AC13	United States	Quality issues	Relay on alternative drug
	Furosemide Tan et al. (2017)	C03CA01	Canada	Slow production	Appropriate alternatives
Anti-coagulants	Heparin Benge and Burka (2019)	B01AB/B01AC	United States	African Swine fever	Compromise alternative therapy
Analgesics	Oral morphine, injectable morphine, hydromorphone, fentanyl Clark et al. (2020)	N02AA01 N02AA03 N01AH01/N02AB03	United States	Opioid epidemic quality issues	Switch to other dosage forms
	Salsalate 500 mg Dave et al. (2018)	N02BA06	United States	Unknown	Price increase
Anesthetics	Midazolam Choo and Rajkumar (2020)	N05CD08	United States	Pandemic, increased demand	May put patients’ health in severe condition as both medicines are for ventilator patients
	Propofol Choo and Rajkumar (2020), Siow et al. (2020)	N01AX10	United StatesSingapore	Pandemic, increased demand	
	Bupivacaine Sun et al. (2020)	N01BB01	United States	Unknown	Used of preservative-free isobaric bupivacaine and adjuvants cause increased side effects
Psychotropic medicines	Lorazepam, Diazepam Clark et al. (2020)	N05BA06 N05BA01	United States	Manufacturer shortage	Use alternative drugs
Anti-epileptic drugs	Topiramide Benhabib et al. (2020)		France	Unknown	Compromise effects with an alternative drug
Vaccines	Hepatitis B, pediatric/Adult Hepatitis A, Zoster vaccines Liang and Mackey (2012)	J07BB04 J07BC02 J07BK02	United States	Unknown	Online purchasing
Radiopharmaceuticals	^99^MO Ballinger (2010)		United Kingdom	Cost raise	Rest of the reactors had to struggles to ensure continuity of supply
	Molybdenum 99				
Electrolyte nutritional support	Amino acid preparations Clark et al. (2020)	B02AA/B05BA01 B05BB02	United States	Hurricane Maria, 2017	Alternative therapies with chances of medication error
	Electrolytes Ventola (2011)			Quality issues	
	Parenteral nutrition therapy Holcombe et al. (2018)	B05BA	United States	Production interruption, quality issues	Component shortage impacts all stakeholders, especially patients up to death
Hormonal drugs	Norepinephrine Donohue and Angus (2017), Vail et al., (2017), Siow et al. (2020)	C01CA03	United States	Production interruption	Increasing mortality, leading to septic shock
			Singapore		
Musculoskeletal agents	Atracurium Siow et al. (2020)	M03AC04	Singapore	Unknown	Alternatives with side effects
Pediatric drugs	IV Sodium bicarbonate Visage et al. (2019)	B05CB04/B05XA02	United States	Unknown	Delay in treatment
Anti-diabetic drugs	Insulin Elbagir et al. (1995)	A10A	Sudan	Manufacturing interruption	Dose reduction, increase times of injection
Oncology drugs	Cytarabine Ventola (2011)	L01BC01	United States	Raw material shortage and quality defects	Compromised and delayed treatment
Anti-spasmodic agents	Papaverine Ricci et al. (2016)	A03AD	Israel	Unknown	Microsurgical lefts had to select other agents with less clinical evidence

## Causes of Drug Shortage

**FIGURE 2:**
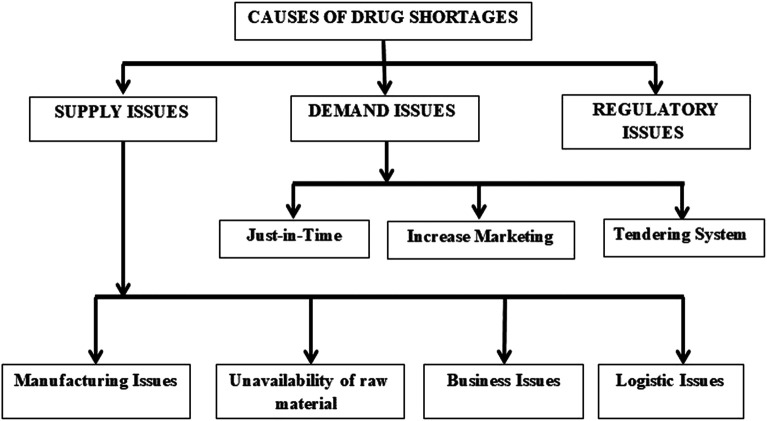
Causes of drug shortages.

There are various reasons for drug shortages, depending upon the type of drug (Mazer-Amirshahi et al., 2014). Overall, drug shortage causes can be classified as supply issues, demand issues, or regulatory issues, as shown in Figure 2. The causes of drug shortages are multifactorial. For example, manufacturing problems, financial pressures, shortage of raw materials, and just in time inventory are found as essential causes of medicine shortages in developed countries of the EU, United States, Saudi Arabia, and developing countries like Pakistan, Fiji (Malik et al., 2013; Hedman, 2016; Zu’bi and Abdallah, 2016; Schwartzberg et al., 2017; Walker et al., 2017; Jenzer et al., 2019).

**FIGURE 3:**
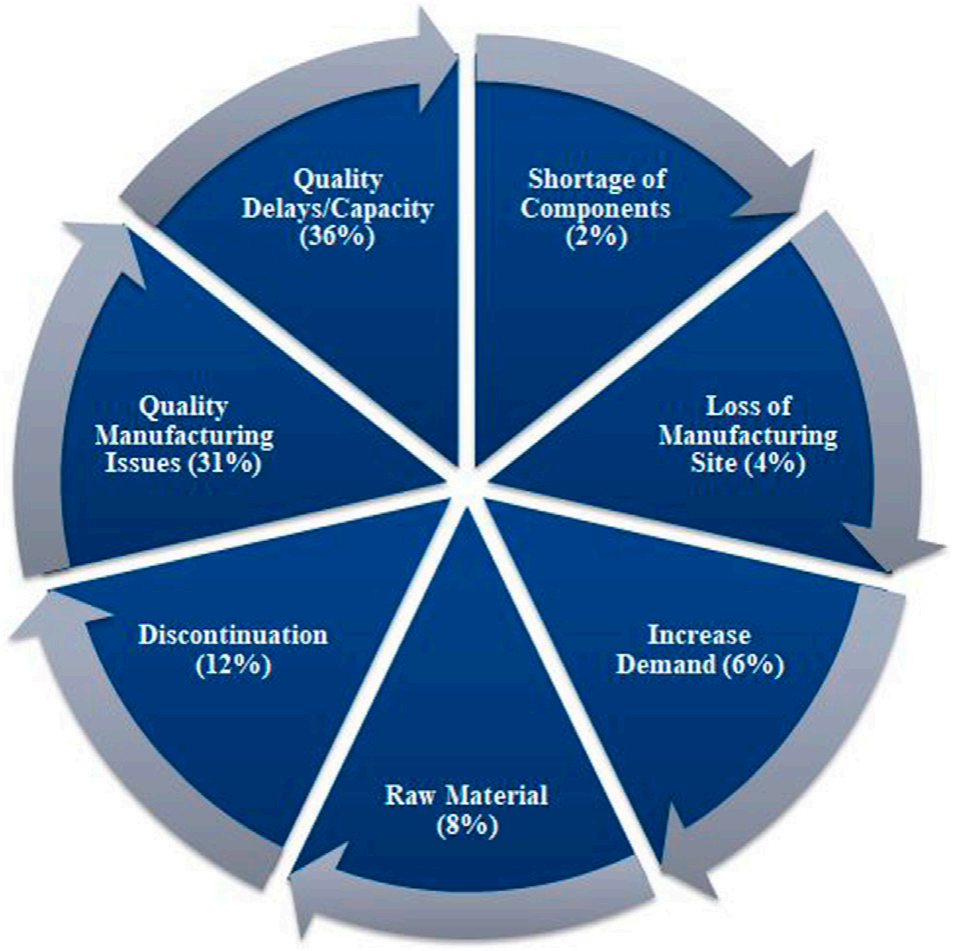
Reasons of drugs in shortages in 2012 reported by University of Utah Drug Information Services (UUDIS) (Dill and Ahn, 2014).

**FIGURE 4:**
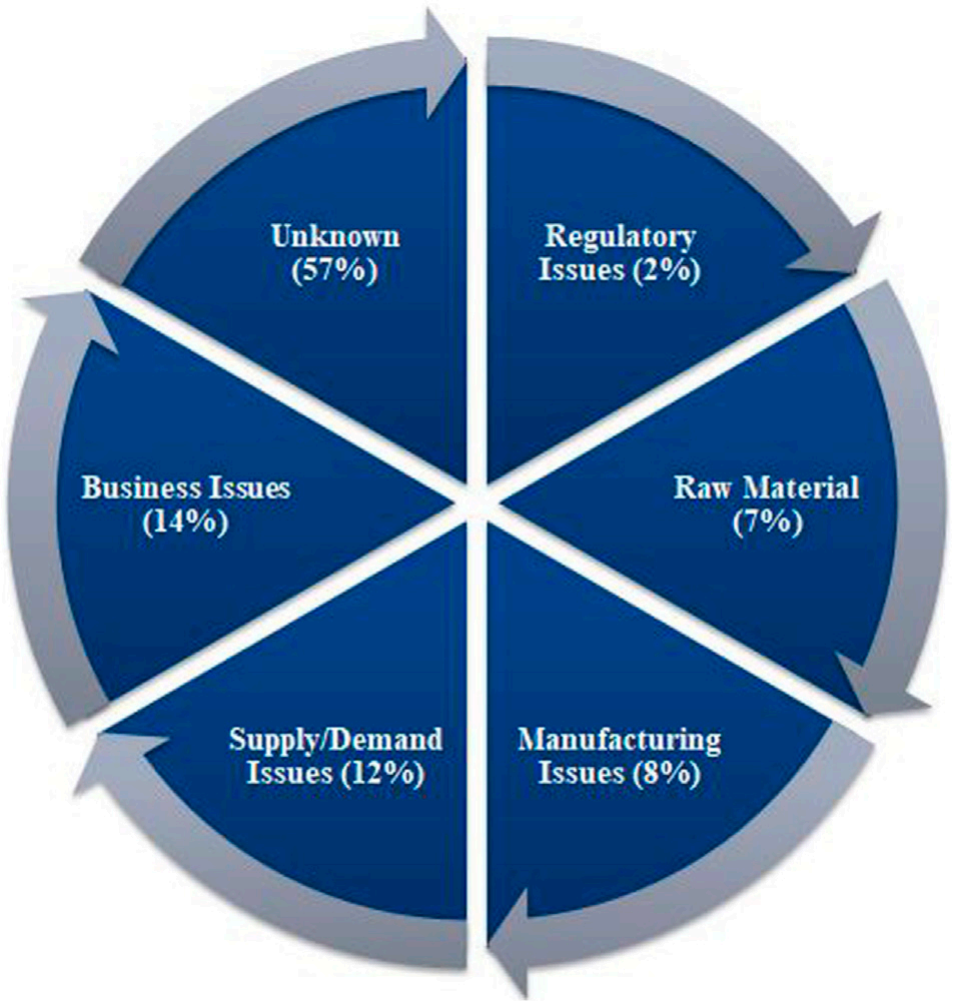
Reasons of drugs in shortages in 2020 reported by University of Utah Drug Information Services (UUDIS) (Pharmacists, 2020).

According to the UUDIS, the quality delays and capacity issues were most dominant in the United States, in 2012 followed by quality manufacturing issues and discontinuation (Figure 3) (Dill and Ahn, 2014), whereas a more significant percentage of reasons was unknown, in 2020, followed by business issues and supply/demand issues (Figure 4) (Pharmacists, 2020). In addition, the 2019 EAHP survey found that the global shortage of APIs, manufacturing problems, and supply chain issues dominated most (Miljković et al., 2020a).

**FIGURE 5:**
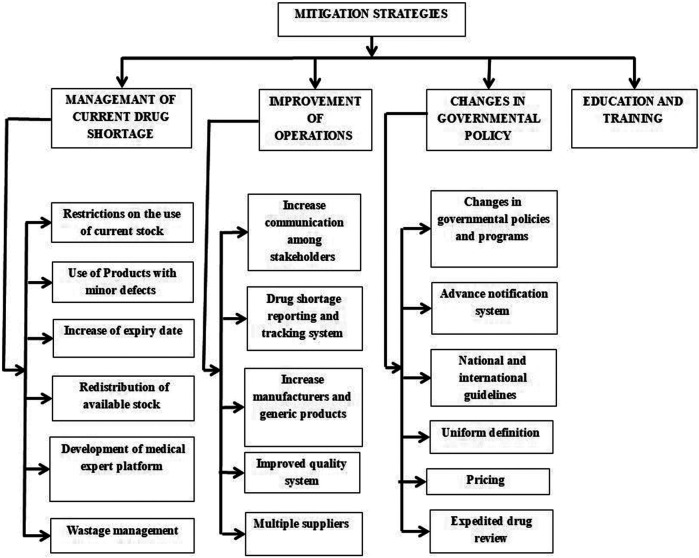
Mitigation strategies.

### Supply Issues

Supply issues mean manufacturers are unwilling or unable to produce enough medicines to satisfy the demand. It can be classified into manufacturing problems, unavailability of raw materials, business reasons (economic reasons like low-profit margin, low market size, cost raise of raw materials, capacity constraints), and logistic problems (supply chain issues) (Dill and Ahn, 2014; Bogaert et al., 2015; Organization, 2015; Alsheikh et al., 2016; Fox and Tyler, 2017; Walker et al., 2017; Alruthia et al., 2018; Fox and McLaughlin, 2018; Pharmacists, 2018; Videau et al., 2019b; Phuong et al., 2019). In high-income countries, these causes have been controlled to some extent, but in the low and middle-income countries, financial burden affects manufacturing quality and capability, medicines availability, affordability, and drug supply chain quantification (Malik et al., 2013; Walker et al., 2017).

#### Manufacturing Issues

The manufacturing issues include quality problems and competing priorities. The EMA is associated mainly with medicine shortages due to manufacturing issues (Hedman, 2016; Schwartzberg et al., 2017). Studies done in Finland, Saudi Arabia, Canada, and the EU mentioned manufacturing issues, the most promising ones (Alsheikh et al., 2016; Heiskanen et al., 2017; Unit, 2017; Videau et al., 2019b). Middle-income countries like Brazil and Venezuela cited shortages of injectable products with disregarded quality due to decreased incidence of some diseases and the availability of other valued alternatives (Acosta et al., 2019). Whereas low-income countries like Pakistan reported manufacturing issues due to lack of regulatory policies and financial pressure (Malik et al., 2013; Fatima and Khaliq, 2017).

1) Quality problems

The quality problems are the most frequent reason for the drug shortage found during daily quality checks and inspections after manufacturing, even after being released to customers. The quality problems usually lead to voluntary recall when found after medicine dispatching in the market. Such issues occur due to microbial contamination (bacteria/fungi), endotoxin, tablet disintegration, particulate matter (glass, metal, fiber, foreign matter) in vials, precipitate formation, or unexpected reaction between products and containers. Good Manufacturing Practice (GMP) violation can also be classified as a quality issue, as those violations may cause quality defects, and ultimately shortages (Dill and Ahn, 2014). In the United States, 67% of drug shortages were due to quality issues in 2012, and the trend was found the same in 2013 (Gray and Manasse, 2012; Mazer-Amirshahi et al., 2014; Alsheikh et al., 2016). The United States FDA warned six renowned sterile product manufacturers for violating the current Good Manufacturing Practices in 2014 (Mazer-Amirshahi et al., 2014).

2) Competing priorities

When a manufacturer can produce several products, these products compete for raw materials, manufacturing lines, and markets. Therefore, manufacturers have a lower motivation to invest or produce medicines with low profitability, such as generics and injectable products, which require a stricter quality environment for manufacturing compared with those medicines with higher profitability, as found for generic products in the United States (Dave et al., 2018) and injectable products in Brazil (Acosta et al., 2019).

#### Unavailability of Raw Material

Drug shortages may occur when there is a problem in the supply of raw materials. It could be a shortage of active pharmaceutical ingredients (API), excipients, or packaging materials (Dill and Ahn, 2014). India and China are the important active pharmaceutical ingredient suppliers for almost all economic levels. Unavailability of raw materials could be due to political turmoil, armed conflicts, animal disease, trade disputes, environmental conditions, degradation during transport, or low plant yield as a source of material from the source country (Ventola, 2011; Coustasse et al., 2020). In 1998, there were shortages of many drugs due to Hurricane George in Puerto Rico (Gu et al., 2011; Rinaldi et al., 2017).

In the COVID-19 pandemic, shortages of active pharmaceutical ingredients (APIs), excipients, and drugs occurred worldwide. As a result, the countries (India, China, United States) the producers of APIs stopped supplying some APIs to other countries that led to the global shortage of many drugs. Moreover, many other challenges, including shortage of packing material, disrupted transport, delayed shipping, delayed customer clearance, restricted import-export of APIs and drugs throughout the world (Ayati et al., 2020; Badreldin and Atallah, 2021). Moreover, in the COVID-19 pandemic, shortages of API were also found in the United States (Coustasse et al., 2020). When a sole supplier provides the API and excipients of any product, any problem with the supplier may lead to a medicine shortage (Ventola, 2011). Therefore, a product with at least three suppliers for materials is usually considered desirable (Hedman, 2016). The unavailability of raw materials was an important reason for drug shortages in Saudi Arabia (Alsheikh et al., 2016), Canada (Videau et al., 2019b), and Pakistan (Atif et al., 2019).

#### Business Issues

Business issues are economic factors, including little profit margin, small market size, consolidation, absence of maintenance, and low procurement ability (Dill and Ahn, 2014; Dranitsaris et al., 2017).

In high-income countries, drug shortages frequently occurred due to business issues. The low market price is the leading cause of generics shortages. Some manufacturers found it difficult to cope with a branded counterpart and upgrade their infrastructures to comply with GMP requirements. Such manufacturers maintained the expensive high-quality standard for their low-profit generic products and left the market, leading to the plant's closure, i.e., the shortage of generic injectable products in the United States (Dill and Ahn, 2014). Even complying with GMP requirements is also costly for some generics manufacturers (Alsheikh et al., 2016; Dave et al., 2018). The absence of industrial plants and other facilities maintenance leads to inefficiency, less production capability, and ultimately drug shortages. Sometimes, different companies combine their manufacturing plants for common drugs to minimize costs and gain significant benefits. Consolidation of manufacturers results in a reduction in the number of manufacturers (Schweitzer, 2013). For a supply chain with only a few manufacturers, flexibility may become an important issue, and the risk of drug shortage will increase (Dill and Ahn, 2014; Emmett, 2019). Drugs with small market size, e.g., orphan drugs, are vulnerable to the shortage as manufacturers rarely prefer manufacturing such drugs (Mazer-Amirshahi et al., 2014). For example, GlaxoSmithKline pulled the Lymerix vaccine off the market as the demand decreased (Gu et al., 2011). Studies in low and middle-income countries reported procurement processes as causes of shortages, e.g., Kenya, Fiji, Pakistan, and Sub-Saharan (Malik et al., 2013; Walker et al., 2017). The drug procurement process needs a specific period, but poor coordination among the departments delayed the procedures, resulting in a shortage. In this situation, local drug purchasing at increased prices puts a financial burden. Still, a further delay of the procurement process beyond the bid validity period leads to increased costs for bidders at quoted rates, worsening the situation (Herath et al., 2011). For example, the delayed procurement process was involved in the antimalarial drug stock-out in Kenya (Malik et al., 2013).

#### Logistic Issues

Logistic issues, including transportation issues and drug supply chain management incompetency, are the causes of drug shortages. The transportation problem is usually due to horrible weather, bad traffic, and natural disasters (Dill and Ahn, 2014; Mazer-Amirshahi et al., 2014). In addition, the United States FDA reported logistical issues as an important reason for the recovery of disruption (Administration UF, 2020).

### Demand Issues

Demand issues include just-in-time inventory and increased marketing (average growth demand, outbreaks (Alsheikh et al., 2016), epidemic, and seasonal demand). It may be predictable or unpredictable. A well-established system can predict the shortage caused by just-in-time inventory, average demand growth, and seasonal demand; however, outbreaks, epidemics, and disasters are unpredictable (Dill and Ahn, 2014; Fox and Tyler, 2017; Walker et al., 2017; Phuong et al., 2019). Moreover, in low and middle-income countries, irrational use of medicines, lack of patients’ education, and prescription adherence lead to medication wastage, compromised outcomes, and increased demand (Walker et al., 2017). Other causes include prescribing practices and unethical medicines promotion by pharmaceutical companies (Malik et al., 2013).

#### Just-In-Time Inventory

Just-in-time (JIT) inventory system is a management stratagem that aligns fixed raw materials/drugs from suppliers directly with the currently scheduled requirement. In the circumstances of a low financial budget, stakeholders purchase a fixed quantity of stock for a fixed duration that fulfills only the current needs of the pharmacy/institution without any backup plan. It is a widespread strategy to run the system with minimum cost but with a greater risk of drug shortage because of no buffer stock reported in high-income countries (Ventola, 2011; Alruthia et al., 2017; Emmett, 2019).

#### Increased Marketing

Increased marketing of a new product or an older one may be predictable or unpredictable. Predictable marketing increases the focus of professionals and the public towards a product. Unethical/uncontrolled marketing strategies are used chiefly in low-income countries where drug-related policies are rare (Abdelrahman et al., 2016). A study performed in Pakistan found that pharmaceutical companies’ unethical marketing of Artemether/Lumefantrine resulted in stock-out of Sulphadoxine/Pyrimethamine (Fansidar) and Chloroquine (Malik et al., 2013). Increased marketing of particular medicines for rare diseases with more incredible physicians’ benefits leads to a significant supply and demand gap (Hedman, 2016).

An unpredictable increase in demand may occur due to an outbreak, natural disaster, fire, or other accident. In such a situation, drug shortage may occur because the lead time of manufacturing is longer for medicines, and sparing lines for manufacturing specific medicines is complex (Dill and Ahn, 2014). For example, saline bags were in shortage in Hurricane Maria in Puerto Rico (Emmett, 2019), and Tamiflu for pediatric use was in short supply in pandemic H1N1 in 2009 (Gu et al., 2011). Such disasters, in return, could cease manufacturing (Dill and Ahn, 2014).

The demands of some medicines depend upon seasons. For example, the demands of cough syrup, pediatric antipyretic syrup, antihistamines, and anti-asthmatic drugs increase in winter. Seasonal demand is often predictable, but it is still a risk factor for drug shortage. For example, Oseltamivir shortage occurred when seasonal influenza attacks (Mazer-Amirshahi et al., 2014).

#### Tendering System

Tendering system caused drug shortages in the United States and EU. Awarding a drug tender to a single supplier would put it at high risk of shortage. Moreover, the low pricing of medicines through tendering compelled some manufacturers to leave the market, leading to the loss of competition and rising prices. The group procurement also caused drug shortages as it changed the purchasing pattern (Dranitsaris et al., 2017).

### Regulatory Causes

Drug regulatory authorities are accountable for effective drug regulation to safeguard drug quality, safety, efficacy, and appropriateness/accuracy of drug information accessible to the public. Still, their compromised roles lead to many problems, including drug shortage. Some of the regulatory issues found in low and middle-income countries are the inflexibility in regulatory processes, lack of policies, and unavailability of communication among stakeholders. These events also included the lack of implementation of essential drug lists that led to the disruption of supply chain management in multiple ways (Malik et al., 2013). A study in Jordan found regulatory issues as one of the four causes of drug shortage (Gu et al., 2011; Bogaert et al., 2015; Zu’bi and Abdallah, 2016). Many studies also mentioned unknown causes of drug shortage due to the unavailability of regulations from the government (Bogaert et al., 2015).

The safety and efficacy standards implemented by United States FDA compelled the entry of new manufacturers into the market, marketed drugs, and some old drugs to review their qualifications, which will take a long time, ultimately leading to the shortage of many drugs (Mazer-Amirshahi et al., 2014). Introduction of new guidelines, changes in drug use guidelines or therapy management guidelines from the government, such as new indications for a drug, changes in therapeutic character, can change supply and demand, resulting in a shortage. For example, changes in CDC guidelines in classifying children to the age range of 6–59 months resulted in a shortage of pediatric flu vaccines in 2006 provided by the solo manufacturer (Ventola, 2011). United States and EU have introduced many advanced initiatives to optimize the drug supply chain. One of them is the Falsified Medicines Directive (FMD) by the EU to keep counterfeit and quality compromised medicines away from the drug supply chain. Though this policy effectively keeps fake medication away, the implementation process caused medicine shortages because the system needed time to improve the quality up to the mark (Bouvy and Rotaru, 2021). The EU (European Union) implemented new GMP (Good Manufacturing Procedure) regulations in 2013 to control and ensure better quality. However, these new guidelines, especially the one related to purchasing and processing raw materials, may delay the production procedure and cause medicines shortages to a certain extent (Academy, 2013).

Al Ruthia et al., in 2018 found that poor drug supply chain management was the main reason for drug shortage in large hospitals of Saudi Arabia (34). The absence of a uniform definition is one of the main issues from the regulatory side. It created gaps to judge the severity of the problem and diminished the mitigation strategies (Kaakeh et al., 2011; Klobuchar, 2011).

## Impact of Drug Shortage

The drug shortage affects all stakeholders, especially patients/consumers, in economic, clinical, or humanistic aspects (Dill and Ahn, 2014; Phuong et al., 2019). In developed countries, due to policies, guidelines, associations, and platforms, the problem has been managed to some extent, so the level of impact is not so high but still alarming. It will remain until the management of this issue globally; for example, studies cited the out-of-pocket cost in the United States, Canada, Europe, Australia, and South Africa. On the other hand, most institutions in LMICs have their strategies, but the government introduces no policies to tackle the vast impacts (Hayes et al., 2014; Pauwels et al., 2014; Perumal-Pillay and Suleman, 2017; Lukmanji et al., 2018; Phuong et al., 2019).

### Economic Impacts

Drug shortages usually result in an extra cost or budget for different stakeholders, especially patients, at all economic levels. In high-income countries, the stakeholders are aware of drug shortages, and the suppliers have to manage unavailability of raw materials through additional operations. The retailers have to purchase a many short-supplied drugs with increased prices or purchase expensive alternative brands, start compounding or logistic modifications. The hospitals have to put extra costs to manage the shortage, such as purchasing costly brands, excess inventories, and awareness programs to deliver staff knowledge. Studies estimated $200 million in purchasing expensive alternatives (“Drug Shortage Cost United States Care Providers” 2011) accompanied by additional labor costs of $359 million (Kacik 2019) in United States hospitals due to drug shortage annually (Fox and Tyler, 2013; Dill and Ahn, 2014; Fox et al., 2014; Mazer-Amirshahi et al., 2014; Costelloe et al., 2015; Hughes et al., 2015; Food and Administration, 2019; Phuong et al., 2019). On the other side, medicine price increases after a drug shortage, especially for lower-priced generics, medicines with a solo manufacturer, unapproved medicines, and orphan drugs (Fox and Tyler, 2017; Dave et al., 2018). An increase in medicine price is also an illegal practice in the grey market that stocks up a large share of medicine in advance and provides them at a higher price to customers in short supply. For example, Cytarabine was in shortage for $12, but it was sold on the grey market with an increased price of $900 (Gu et al., 2011). The out-of-the-pocket cost of patients increased as they have to purchase costly brands, expensive alternatives, costly compounded medicines, pay more for a prolonged duration of therapy, extended hospital stay, and compromised treatment, as cited in studies done in Canada, the United States, Europe, and Australia (Phuong et al., 2019). In a study done on 1,650 oncology pharmacists in the United States in 2011, it was found that 93 participants agreed that the drug shortage resulted in altered treatment or delay in chemotherapy administration, whereas 85% of participants agreed that the shortage led to increasing cost of the regimen (Dill and Ahn, 2014). Drug shortages also lead to online purchasing from illegitimate vendors that are difficult to differentiate for the consumers. In addition, such purchasing increased the financial burden for patients as they are available at increased prices than in pharmacies (Jackson et al., 2012; Fittler et al., 2018; Koenraadt and van De Ven, 2018).

Very few studies have been done in low and middle-income countries, only reporting increased out-of-pocket expenses as the economic impact of drug shortages (Fatima and Khaliq, 2017; Walker et al., 2017; Acosta et al., 2019). Management of medicines shortages depends strongly on the health care system with a well-managed drug supply chain and adopted health care model. The high-income countries have adopted the Beveridge Model (Spain, New Zealand, Cuba, Hong Kong), Bismarck Model (Germany France, Belgium, Netherlands, Latin America), and National Health Insurance Model (Canada, South Korea, Taiwan), where patients are relieved from the out-of-pocket costs. However, in the middle and low-income countries, the Out-of-Pocket Model (remote areas of China, India, Africa, and South America) is working, burdening the patients (Kos, 2019).

Importation of medicines in the case of national shortage leads to severe economic impacts burdening the government. In the EU, the import/export criteria are not harmonized, and different countries have different policies to allow the import and export of medicines in shortage. The lack of harmony and the parallel import from EU or extra-EU countries may threaten the medicines’ availability in a country with a ripple effect. The uncontrolled movement of drugs could be worsened by this ripple effect, causing severe economic impacts (Musazzi et al., 2020; Vida et al., 2016).

### Clinical Impacts

Clinical outcomes of drug shortage have been reported in the majority of studies in developed countries. It included alterations in treatment, inferior treatment, prescription inaccuracies, dispensing errors, administration errors, delayed or denied treatment, prolonged hospitalization, adverse drug interactions, and even death reported by studies done in the United States, Saudi Arabia, Europe, Australia, Canada, United Kingdom (Becker et al., 2013; Mclaughlin et al., 2013; Dill and Ahn, 2014; Fox et al., 2014; Alsheikh et al., 2016; Mclaughlin et al., 2017; Rinaldi et al., 2017; Schwartzberg et al., 2017; Walker et al., 2017; Dave et al., 2018; Phuong et al., 2019). A survey conducted in North Carolina, South Carolina, Georgia, and Florida reported that drug shortage caused a significant percentage of medication errors in patients leading to compromised health outcomes and increased patient burden creating an unsafe situation for both staff and patients (97). Alfuzosin replaced Tamsulosin which was in short supply, but Alfuzosin increases QT interval (Hsia et al., 2015; Shaban et al., 2018). The shortage of antimicrobial drugs is critical as their shortages leading to delayed treatment, chronic infection, and other deadly outcomes (Fox et al., 2014). In addition, some drugs in the grey market become substandard with time as stored in non-optimal conditions and lead to compromised health outcomes (Fox et al., 2014; Zwaida et al., 2019).

The available online medicines may have quality problems. Many research studies found that online purchasing occurs in high-income countries like Malta, the United Kingdom, Netherlands, with an increased risk of counterfeit medicines and increased drug prices than local purchasing (Jackson et al., 2012; Fittler et al., 2018; Koenraadt and van De Ven, 2018).

Drug shortages lead to inappropriate alternatives in prescription, compromised health, prolonged hospital stay, readmission, morbidity, and mortality in developing countries (Uganda, Fiji, Zambia, Nigeria, Egypt) (Malik et al., 2013; Walker et al., 2017). Scientific studies have proven that the interrupted treatment due to the drug shortage for antiretroviral therapy (ART) led to substandard outcomes, accumulations of drug resistance mutations, and treatment failure (Meloni et al., 2017). For some critical medicines, their shortage will result in the cancellation of surgery. For example, the shortage of protamine sulfate will lead to the cancellation of heart surgery. Surgery cancellation may worsen the disease, prolong hospital stay, and expose hospital-acquired infections (Burki, 2017; Khan, 2019). More seriously, the shortage of some medicines may lead to higher mortality. Due to chemotherapeutic drug shortage, the mortality rate was high, but essential drugs, including antibiotics, Phytonadione, electrolyte solutions, analgesics, and opioids, were also involved (Mazer-Amirshahi et al., 2014). Drug shortage also increased online purchasing of counterfeit products in middle-income countries (Yin et al., 2016; Huang and Xu, 2017).

### Humanistic Impacts

The consequences of treatment or disease on patient quality of life (QOL) are humanistic outcomes, e.g., patient satisfaction with treatment outcomes and hospital services.

Drug shortages resulted in varied humanistic impacts on patients, and health-care professionals noticed many types of research in developed countries (Dill and Ahn, 2014) and developing countries. Studies in the United Kingdom, Canada, and the United States found that shortages usually give rise to patient complaints, frustration, anger, dissatisfaction, decreased adherence, and psychological effects. Patients also complain of traveling anxiety about other medicines, and face delayed surgery (Schwartzberg et al., 2017; Dave et al., 2018; Phuong et al., 2019). The drug shortage situation makes physicians dissatisfied, stressed, exasperated, lost patients’ trust, even threatening them (Hedman, 2016; Rinaldi et al., 2017; Walker et al., 2017). Physicians have to choose patients for receiving limited available drugs or are forced to select alternative therapy (Russell et al., 2017). The situation becomes more critical for cancer patients (Dill and Ahn, 2014; Hedlund et al., 2018).

A few studies in LIMCs reported that professionals face increased frustration, threat, violence, negative workplace situation. They have to put extra effort and time into managing the shortages. The patients lost trust in the hospitals and concern about alternates effects (Walker et al., 2017; Phuong et al., 2019).

## Mitigation Strategies

Drug shortages were studied well in the United States (South and North America), European Union, Oceania countries (Fiji, Australia). In contrast, few related studies were found in Asia (Saudi Arabia, Pakistan, China, Iran, Iraq, Jordan, and Israel) and Africa (Kenya, South Africa, Egypt, and Uganda). In the United States and European countries, extensive work has been done to implement policies for drug shortages mitigation, but there is an extensive research gap in the rest of the world and policies (Acosta et al., 2019).

Different strategies are proposed in most of the high-income and some middle-income countries to cope with drug shortages. International and national organizations, including the World Health Organization (WHO), the International Pharmaceutical Federation (FIP), American Society of Health-System Pharmacist (ASHP), and the European Association of Hospital Pharmacists (EAHP), are involved a lot in taking initiatives, providing information and guidelines to mitigate the drug shortage situation as shown in Figure 3 (Fda, 2013). Simultaneously, many approaches were proposed by the United States, European countries, Canada, Australia, China, etc., However, this problem is still on the ground and has been ignored in most low and middle-income countries, so there is a need for ever-growing, universal, and updated versions of the strategies to put the issue on the knee internationally.

In hospitals, the health care team uses following strategies managing drug shortages: 1) informing prescribers and recommending them alternative agents, 2) contacting other suppliers for the short medicine, 3) investigating supply restoration and planning, 4) substituting the prescribed medication and 5) updating the formulary. However, in the community pharmacies, community pharmacists and working staff use to manage the drug shortages by adopting strategies: 1) management of current shortages, 2) contacting the authorized supplier, 3) contacting other pharmacies, and 4) suggesting an alternative treatment to the patient (Tan et al., 2016; Panic et al., 2020).

### Management of Current Drug Shortage

**1) Restrictions on the use of current stock.** When there is limited stock of some medicine in a medical institution and no supply for an unknown period, the institution should limit the stock to specific patients. Some modelling methods or mechanisms can be used to give priority to patients, like the A4R framework. Such modelling methods prioritize patients with restorative therapy, pediatrics patients, cancer patients with no alternative available, patients in clinical trials, and patients on treatment regimens with authentic survival benefits (Valgus et al., 2013). The strategy is crucial for the drugs with rarely marketed generics, anticancer drugs, and emergency drugs reported by health professionals of different Fiji institutions. It will also give time to staff to search for other alternatives (Walker et al., 2017).
**2) Use of products with minor defects.** Products with minor defects like particulate matter, the viable substance, can be used after proper handling in case of shortage. The United States FDA has adopted the strategy, and they allowed the use of medicines with improper packaging, labelling defects, glass particles in an injectable (filter before use), and non-registered drugs after risk evaluation or proper handling to overcome the shortage (Mazer-Amirshahi et al., 2014).
**3) Increase of expiry date.** When shortage occurs, drugs with near expiry dates can be used by extending the printed expiry date. For example, the United States FDA has used this strategy and extended the expiry date of coral snake antivenin, which was discontinued by the manufacturer (Mazer-Amirshahi et al., 2014).
**4) Redistribution of available stock.** In the drug shortage situation, the manufacturer is responsible for the redistribution of currently available supply in a consistent manner among different areas or different institutions. Good communication and transparency among stakeholders with an adequate reporting system are required to achieve an efficient and equitable redistribution of the available quantity of shortage drugs (Fox et al., 2014; Schwartzberg et al., 2017).
**5) Development of the medical expert platform.** A medical expert’s platform at the institution level will help handle the shortage of drugs proactively. The experts’ team should comprise pharmacists, physicians, and nurses. The platform should provide information about future shortages, manage current shortages, tackle and review alternative treatment, stock alternatives, and guidelines for restriction on medicine use through enhanced communication within the institution and other institutions (Dill and Ahn, 2014; Rhodes et al., 2016). The United States has established a platform that conducts institutional level analyses and forecasts drug shortages (Valgus et al., 2013; Modisakeng et al., 2020). Moreover, compounding low-risk medicines in severe shortage can be used when there is no other option, but high-risk medications could lead to severe adverse effects (Lyon, 2012; Badreldin and Atallah, 2021). Similarly, a study found that off-label medicines for cancer patients could only use when there is no other option to treat them (Valgus et al., 2013; Frattarelli et al., 2014).
**6) Wastage management.** In treating chronic conditions, especially oncology and pediatric patients, the dose is small (e.g., 12 mg/250 mg containing vial), so the leftover drug goes to waste due to short shelf life. To utilize the leftover drug in vials, United States hospitals’ oncology departments schedule their chemotherapy by clustering the patients on a specific date. This strategy is also adopted in the anaesthesia department through a double syringe technique (Khan, 2019; Rowe et al., 2020). The benefits of this strategy are multidimensional, including overcoming the shortage, preventing drug wastage, and educating the caregivers about the shortage and its management (Mazer-Amirshahi et al., 2014; Rowe et al., 2020).

### Improvement in Operations

**1) Increase communication among stakeholders.** Communication among regulatory authorities and stakeholders at the national and international level is critical in administering drug shortages (Costelloe et al., 2015; Schwartzberg et al., 2017; Walker et al., 2017). Good communications make proactive actions possible, which can manage the issue with minimum loss. It should be accompanied by harmonization and transparency among all stakeholders (Fox and Tyler, 2003; Hedman, 2016; Nixon, 2021). Health Canada started Regulations Amending the Food and Drug Regulations, a reporting system for drug shortages, in 2015 that helped prevent and manage drug shortages by enhancing communication in the Canadian market (Pauwels et al., 2015).
**2) Drug shortage reporting and tracking system.** A reporting and tracking system can report all related aspects of a drug shortage, including its period, drugs involved, frequency, duration, causes, impacts, managing strategies, and predict future shortages. Such a system should be present at every institution (Giammona et al., 2020) as done by the Drug Shortage Program (DSP) developed by the FDA in 1990 (Dill and Ahn, 2014; Said et al., 2018) and Texas Medical Board (Mazer-Amirshahi et al., 2014). The databases and public websites are found in many middle and high-income countries of the European Union, the United States, and Western Asian countries. Still, they have no information about the impact of shortages and need improvements (Acosta et al., 2019).
**3) Increase in manufacturers for generics.** Some medicines have a higher risk of shortage, such as injectables and generics. High costs caused by strict GMP protocols for injectable medicines and low prices for generics lead to a few manufacturers and a vulnerable supply chain. The incentive pricing mechanism is needed to spur more manufactures to produce these medicines (Alspach, 2012; Panzitta et al., 2017).
**4) Improved quality system.** The quality of a drug is vital for its optimum efficacy, and it can compromise during its manufacturing, storage, transportation, and until the time of administration. The drug shortages rising through recalls due to compromised quality can be prevented through quality assurance and quality control departments (Mazer-Amirshahi et al., 2014; Said et al., 2018). The regulatory authorities should implement strict protocols for manufacturers to comply with quality and reward manufacturers with a sound quality system to encourage more manufacturers to value the quality system (Alspach, 2012; Panzitta et al., 2017).
**5) Multiple suppliers.** Manufacturers should contact more than one supplier for a single raw material (Dill and Ahn, 2014). In evaluating new suppliers, the regulatory authorities also should pay special attention to those medicines currently with only one supplier (Fox et al., 2014). And sometimes, speeding up the reviewing process is needed when there is a higher risk of shortage.

### Changes in Governmental Policy

**1) Changes in governmental policies and programs.** The governments of many countries have introduced various organizations to manage drug shortages by adopting policies and programs. United States FDA established Drug Shortage Program (DSP) to deal with the shortage issue specifically. Some researchers also suggest an upgrading of DSP by focusing on all types of drugs. The developing countries should follow the foot's steps of developed countries in conducting researches, establishing platforms, policies, and guidelines for medicines and their shortages (Babar, 2021). There is a need for a more robust, well-organized, and optimized management system under governmental organizations, as adopted by developed countries, to update, monitor, and control the whole drug supply chain. It should focus on national pedigree laws that will automatically restrict pharmaceutical product distribution to licensed distributors, as seen in the EU through catalogue development (Gu et al., 2011) and price gouging (Gu et al., 2011; Zu’bi and Abdallah, 2016; Nematollahi et al., 2018; Jia and Zhao, 2019), along with penalties for those who will violate the policies (Gu et al., 2011; Dill and Ahn, 2014; Schwartzberg et al., 2017). For this purpose, governmental policies should involve more trained professionals and other medical experts for better procurement, stock evaluation, logistics, and mitigation plans for drug shortages (Bazargani et al., 2014; Hedman, 2016), as found in France (Dranitsaris et al., 2017). In low and middle-income countries, governmental policies and programs are absent. Adopting a robust system will portray a better, viable, and fair picture of the whole chain to manage drug distribution and drug shortage (Alazmi and AlRashidi, 2019).

The pandemic warned the medicines supply chain globally. For example, the United States started to resume home production and stop trading with other countries. This de-globalization strategy decreases the risk of medicines shortages due to disruption of the supply chain outside of the country (Vida et al., 2016; Coustasse et al., 2020; Roehr, 2020); however, it also increases the risk of medicine shortages in countries without pharmaceutical manufacturing.

**2) Advance notification system.** Many countries have adopted an advance notification system like France (2004), Belgium (2006), Spain (2010), Switzerland (2015), and Canada (2017) (Fda, 2013; Dill and Ahn, 2014; Iyengar et al., 2016; Alazmi and Alrashidi, 2019), where it is enforced for the manufacturers to give notice to regulatory authorities in case of any disruption (Schwartzberg et al., 2017; Walker et al., 2017). Still, there is no regulation or penalty for those who do not provide early notification to the health authorities (Gu et al., 2011; Mazer-Amirshahi et al., 2014). United States FDA prevented 170 shortages in 2013 due to the advance notification system (Dill and Ahn, 2014; Fox et al., 2014). A study conducted in Europe and Israel stated that the respective authority usually gets notification two months before shortage (Miljković et al., 2020b). There is no strict regulation and policy for advanced notification by manufacturers in developing countries like Pakistan, but multinational companies rarely notify severe shortages (Atif et al., 2019).
**3) National and international guidelines.** International organizations worked in collaboration and presented guidelines for drug shortage mitigation (Fox and McLaughlin, 2018). WHO gave global mitigation guidelines that included: increased training and global communication among stakeholders, patient-focused care, rising production rates, engagement of Non-Governmental Organizations (NGOs) to provide buffer stock, availability of worldwide reporting systems, an advance notification from manufacturers, reasonable quality control, and feasible medicine pricing (Alsheikh et al., 2016; Miljković et al., 2020c). These guidelines, along with other approaches proposed by the FDA, ASHP, and EMA, act as a foundation for other countries for making national guidelines (Alazmi and AlRashidi, 2019). Many countries also develop guidelines at the national level. Israel Ministry of Health (MOH) put guidelines in which the manufacturers have to send an early notification to MOH about any shortage. It has shared protocols to manage the shortage involving all stakeholders. Each country should develop and upgrade national guidelines covering all necessary aspects of drug shortages (Schwartzberg et al., 2017).
**4) Uniform definition.** Drug shortage is a global event. And it needs a uniform scale to apply it at the international level, cover all stakeholders, and overcome humanistic, economic, and clinical impacts. The uniform definition willbetter picturethis issue’s severity by measuring with standard scales and a better strategy to handle it (Alsheikh et al., 2016; Schwartzberg et al., 2017; Bochenek et al., 2018).
**5) Pricing.** The regulatory authorities should design a viable pricing policy by considering all stakeholders. There should be fair criteria for the manufacturers of low-priced generics to gain appropriate profits. For expensive products like oncology drugs, the price must be placed in an acceptable range of customers (Mazer-Amirshahi et al., 2014; Hedman, 2016; Morgan and Persaud, 2018). A viable pricing policy helps developing countries, keeps stock up to their level, and controls the false scarcity (to increase prices) (Emmett, 2019).
**6) Expedited drug review.** Each country’s regulatory authority reviews the medical products to check whether they comply with the standard and launch new products in time. The lengthy process with a high fee will automatically delay the production and cause a drug shortage. Therefore, one of the critical steps to prevent shortages by the United States FDA in 2012 and 2013 was expediting drug reviews to restore production and introduce new products. Some researchers also suggest that United States FDA can accelerate the review process of abbreviated new generic drug applications and exclude user fees for selected drugs or manufacturer evaluation to boost new market entries (Fox and Tyler, 2017).

### Education and Training

Education and training of all staff involved in the drug supply chain are crucial to tackling the shortage. In addition, using alternatives properly (dose, administration, and side effects), optimizing inventory management, and scheduling stocked-out medicines are critical to mitigating the situation. Many organizations from the developed countries are providing such kinds of information on their websites, such as the Drug Shortage Program (DSP), American Society of Health-System Pharmacists (ASHP), Texas Medical Board, and European Association of Hospital Pharmacists (EAHP) (Dill and Ahn, 2014; Mazer-Amirshahi et al., 2014).

With the help of health professionals, especially pharmacists, developing countries’ regulatory authorities can play their role in educating other professionals and giving awareness to the public (Caulder et al., 2015). There is a need for special training and knowledge in treating pediatric patients, especially neonates (Ziesenitz et al., 2019). Ongoing information and communication provided by clinical pharmacists on drug shortage duration resulted in a promising decrease in medication errors in the Pediatric Intensive Care Unit (ICU) (Hughes et al., 2015).

Public education and awareness through advertisements, campaigns, notices about drug shortages are also essential to correct their perceptions, rebuild their behavior (increase adherence), and trust in the healthcare system for positive outcomes (Ziesenitz et al., 2019). Patients should be aware of the availability of drugs in nearby clinics rather than general hospitals as increased patient flow to public hospitals causes drug shortages (Alsheikh et al., 2016; Alazmi et al., 2017; Walker et al., 2017). Patients want to know about their surgeries and drug choices (Hsia et al., 2015), so the decision-making process should include their preferences when the drug is unavailable (Orlovich et al., 2020).

## Discussion and Conclusion

Drugs shortage is a multifactorial issue and affects globally. It has gained plenty of attention in most high-income countries with the development of many associations, governmental agencies, platforms, and policies (Acosta et al., 2019). On the other hand, only a few research studies are found in some middle-income countries, and there is a scarcity of studies in low-income countries (Schwartzberg et al., 2017). The drug shortage situation also varied among different countries. In high-income countries, almost all classes of medicines are affected and found short in different periods. This could be due to the increased research studies that highlighted the issue. The high-income countries have developed comprehensive strategies to manage this shortage issue to some extent, which are the example for the rest of the world. The two draft definitions by the WHO (Organization, 2017) and the adaptation of uniform definition in the EU (Musazzi et al., 2020) are the milestones in managing drug shortages.

However, in low and middle-income countries, the issue is rising. In low-income countries, the availability and affordability of essential medicines are still the priority issues. To tackle the drug shortages, evidence from all countries, especially low-income countries, is needed to compare and develop global mitigation strategies.

International regulatory authorities need to cooperate in developing a global mitigation plan with a uniform definition. The global mitigation plan must include strategies for low and middle-income countries. Moreover, at the national level, low and middle-income countries should take steps to develop a proactive system for advance notification, reporting, and tracking of drug shortage information. Effective policies should be implemented to: develop a robust supply chain, motivate manufacturers for valued quality systems, and manufacture those medicines with a higher risk of shortage. Special attention is needed on the clinical side by training health professionals and educating the public to minimize health loss. In conclusion, it is essential to involve all the stakeholders at the national and international levels to cope with this global threat at all economic levels.
